# Progesterone and Cortisol Levels in Blood and Hair of Wild Pregnant Red Deer (*Cervus Elaphus*) Hinds

**DOI:** 10.3390/ani10010143

**Published:** 2020-01-16

**Authors:** Domenico Ventrella, Alberto Elmi, Martina Bertocchi, Camilla Aniballi, Albamaria Parmeggiani, Nadia Govoni, Maria Laura Bacci

**Affiliations:** Department of Veterinary Medical Sciences, University of Bologna, 40064 Ozzano dell’Emilia (BO), Italy; domenico.ventrella2@unibo.it (D.V.); alberto.elmi2@unibo.it (A.E.); camilla.aniballi2@unibo.it (C.A.); albamari.parmeggiani@unibo.it (A.P.); nadia.govoni@unibo.it (N.G.); marialaura.bacci@unibo.it (M.L.B.)

**Keywords:** red deer, hind, reproduction, progesterone, cortisol, hair

## Abstract

**Simple Summary:**

The red deer, also known as the royal deer or European deer, is an artiodactyl mammal belonging to the Cervidae family, widely diffused in almost all of continental Europe. At the beginning of autumn in the Northern Hemisphere, the mating season begins. The males of red deer, called stags, are synchronized with the females, called hinds; indeed, at the beginning of the mating season, they show a marked increase in testosterone to match the hinds’ estrus cycle. Gestation lasts about 230 days, so that calves are born in mid to late spring, the most favorable period for their survival. Scientific data on the reproduction physiology of this peculiar species in wild conditions are lacking, including hormonal variations during pregnancy. The present study describes mean levels of two critical hormones, cortisol and progesterone, in both blood and hair of wild pregnant red deer hinds. Correlation analysis confirmed how animals hunted in later phases of pregnancy have higher hair progesterone.

**Abstract:**

The red deer (*Cervus elaphus* L., 1758) is one of the largest deer species in the world. Females are seasonal polyestrous, with negative photoperiod: the increase of the night peak of melatonin determines the secretion of GnRH and, therefore, LH and FSH. To date there is little information regarding the hormonal control during pregnancy for this species; this could be due to the difficulty of sampling wild subjects, while farmed animals’ hormonal concentrations may not reflect the physiology of the animal in a natural state. In this study we evaluated the concentration of cortisol and progesterone, extracted from blood and hair, on 10 wild and pregnant red deer females. Belonging to the population of the Bolognese Apennines (Italy), the hinds were sampled in the January–March 2018 period, according to the regional selective hunting plan. Plasma progesterone (P4) ranged from a minimum of 1.9 to a maximum of 7.48 ng/mL; while hair P4 concentrations varied from 41.68 to 153.57 pg/mg. The plasma and hair cortisol ranges are respectively 0.4–2.97 ng/mL and 0.03–0.55 pg/mg; the only significant correlation was found between hair concentration of P4 and the date of death. The results of this preliminary study represent a small step towards a better knowledge of this species’ physiology during pregnancy.

## 1. Introduction

The red deer (*Cervus elaphus* L., 1758) species, as the majority of the cervid taxa, shows a very deep relationship between its peculiar reproductive physiology and the their living environment [1]. It is widely diffused in almost all of continental Europe; in Italy it can be identified in a large Alpine area that extends from Cuneo to Udine, while in the Apennines the red deer occupies four distinct areas: the first corresponds to most of the mountain territory of the provinces of Pistoia, Prato, Florence, and Bologna, the second to the Tuscan-Romagna Apennines, the third is represented by the Abruzzo National Park together with the neighboring territories and the fourth by the mountain massif of the Maiella. Lately, some specimens have also been found in southern regions, such as Sardinia [2]. The original habitat of the red deer is constituted by wooded areas with clearings or areas of little thick bush, generally in a flat environment or at low altitudes. However, over the years it has successfully adapted to live in different areas, from the heath to the forest of conifers, due to the increasing human competitive pressure [3].

In order to guarantee a good survival rate and growth of the offspring, hinds need to give birth at an appropriate time [4]. In the Mediterranean area, calves are usually born in spring and lactation goes on during summer [1], in order to exploit the most favorable time of the year, implying an extremely high synchronization between male and female reproductive activity during the rutting period. Red deer hinds are seasonally polyestrous, with a mean length of the estrous cycle of 18 ± 7 days and a gestation of approximately 231 days [5]. The reproductive cycle begins in October and if pregnancy does not occur, ovulation continues until March showing up to eight cycles [5]. The red deer, as well as fallow (*Dama dama*) and sika (*Cervus nippon*) deer, is a photoperiodic species: starting from the summer solstice, the decrease in daylight hours causes an increase in the duration of the nocturnal peak in melatonin secretion [6]. This event is responsible for the activation of the reproductive axis, leading to an increased secretion of LH releasing hormone, known as GnRH, and subsequent release of LH and FSH by the pituitary gland [7].

The afore-mentioned physiological events makes melatonin a major coordinator of the reproductive system and the annual rhythm [8]. The same endogenous circannual rhythm has been confirmed in red deer males, called stags, that show highly synchronized testicular cycles with a peak that overlaps the females’ ovulation [5,9,10]. The almost complete suspension in spermatogenesis, during the non-rutting season (February–April), is further proof of such synchronization process [11]. Progesterone (P4) secreted by corpora lutea is necessary for the maintenance of pregnancy [12,13], while the contribution of placental progesterone is still to be assessed. P4 blood concentrations remain high (≅4 ng/mL) for the duration of gestation, and start decreasing one to two days before parturition in order to reach baseline levels after delivery [4,14]. 

In mammals, many other hormones undergo changes in blood concentration during the period of pregnancy, but how these relate to reproductive processes and their effects are poorly understood in wildlife. Cortisol (CORT) is another important hormone, generally used as stress marker also in wild animals [15], but the evaluation of its plasma concentrations is considered as poorly reliable since the hypothalamic–pituitary–adrenal axis is instantaneously activated upon stressful stimuli, such as restraint and blood sampling [16]. This is partially why, in the last years, alternative matrices for hormones’ and other analytes’ quantification have been proposed, including hair and feces, capable of providing different information regarding a longer timespan [17,18,19], also in red deer [20,21] and other frequently hunted ungulates [22]. For example, fecal CORT was proven to increase during late pregnancy with 10-year old females showing higher levels in comparison to five-year old hinds. It is interesting to note that fecal cortisol levels in wild animals increased during the calving period between May and June, potentially because of the stress related to delivery [23]. Despite the interest of the scientific community towards the physiology of reproduction of different species of cervids, there is currently only very few information regarding wild red deer hinds and pregnancy. Studies have been performed on farmed hinds [24], but such data could potentially differ from wildlife animals in light of the strong influences of the environment on such species. This lack of data can be partially imputed to the difficult nature of sampling. Since these animals can only be killed during certain periods of the year according to the local hunting regulation and usually undergo biometric examinations immediately after, collaborations with control centers for the collection of biometric records and hunters may help increasing the chances of sampling [19].

In light of the afore-mentioned reasons, the aim of this research was to analyze and describe hair and plasma levels of progesterone and cortisol from samples collected from pregnant wild red deer hinds killed during the hunting season in the south-western Bologna Apennines (Italy) area. Data were analyzed to highlight potential correlations with the age of the animals and the date of death. Hopefully, this will help gaining more knowledge regarding the reproduction of this wild species and its relationship with the environment.

## 2. Materials and Methods 

### 2.1. Animals and Sampling

Ten (n = 10) pregnant red deer hinds were killed between 26 January and 1 March 2018 in the south-western Bologna Appennines (Italy) according to the regional hunting calendar (Resolution No. 473 of the Emilia Romagna Regional Executive, 10 April 2017). Upon death, hinds were immediately transferred to the pertinent control centers for the collection of biometric records where the samplings were performed. Ages were estimated from analyses of teeth eruption and wear patterns [25]. Pregnancy was confirmed by direct visualization of the uterus and identification of corpora lutea in the ovaries. Blood was collected from the jugular vein in sterile Lithium-heparin tubes, while hair was clipped from the dorsal-caudal region of the animals. All samples were refrigerated (5 ± 1 °C) and transferred in a cooler, within 24 h, to the physiology laboratories (ANFI-ASA) of the Department of Veterinary Medical Sciences of the University of Bologna (Ozzano dell’Emilia, Italy) as previously described [19].

### 2.2. Samples Preparation and Extraction

Upon arrival, blood samples (1 mL) were centrifuged (4000× *g*, 20 min) and stored at −20 °C until analysis for plasma hormone levels. Progesterone and cortisol were extracted by mixing 0.2 mL of plasma with 5 mL of petroleum ether or diethyl ether, respectively, for 30 min on a rotary mixer as described by Bono et al. [26]. The tubes were centrifuged at 2000× *g* for 15 min and the supernatants evaporated to dryness at 37 °C under an air-stream suction hood.

The hair samples were handled and analyzed as previously described [27]. Briefly, hair samples (250 mg) were washed with water and isopropanol in order to remove any organic residue from the surface. Once fully dried, samples were finely pulverized (180 mg) and incubated overnight with 6 mL of methanol for steroid extraction. After centrifugation, methanol was collected and evaporated to dryness under an air-stream suction hood.

### 2.3. Progesterone and Cortisol Radioimmunoassay

The dry extracts were stored at −20 °C until reconstitution in assay buffer for measurement of P4 (20 µL plasma equivalent, 6 mg hair equivalent) or cortisol (40 µL plasma equivalent, 60 mg hair equivalent) by radioimmunoassay; tritiated P4 (30 pg/0.1 mL; 96.6 Ci/mmol; PerkinElmer Inc., Boston, MA, USA) or tritiated cortisol (30 pg/0.1 mL; 94.6 Ci/mmol; PerkinElmer inc. Boston, MA, USA) were added, followed by rabbit anti-progesterone serum (0.1 mL, 1:10,000; antiserum produced in our laboratory) or rabbit anti-cortisol serum (0.1 mL, 1:20,000; produced in our laboratory), respectively. After incubation and separation of antibody-bound and -unbound steroid by charcoal-dextran solution (charcoal 0.25%, dextran 0.02% in phosphate buffer), tubes were centrifuged (15 min, 3000× *g*), the supernatant was decanted, and radioactivity immediately measured using a β-scintillation counter (Packard C1600, Perkin Elmer, USA).

The sensitivity of the P4 assay was 3.87 pg/tube, the intra- and inter-assay coefficients of variation were 5.7% and 9.4%, respectively. The sensitivity of the cortisol assay was 5.5 pg/tube, the intra- and inter-assay coefficients of variation were 4.9% and 8.7%, respectively.

Cross reactions of other steroids with antiserum raised against P4 were progesterone (100%), 11α-hydroxyprogesterone (9.7%), 5α-pregnan-3-20-dione (4.4%), 17α-hydroxyprogesterone (1.5%), 20α-hydroxyprogesterone (0.3%), cortisol (0.05%), testosterone, and 17β-estradiol (<0.001%).

Cross reactions of various steroids with antiserum raised against cortisol were cortisol (100%), cortisone (5.3%), 11α-deoxycortisol (5.0%), corticosterone (9.5%), 20α-dihydrocortisone (0.4%), prednisolone (4.60%), progesterone, and testosterone (<0.001%).

In order to determine the parallelism between hormone standards and endogenous hormones in red deer hair, a pool sample containing high concentrations of cortisol and progesterone was serially diluted (1:1–1:8) with assay buffer. A regression analysis was used to determine parallelism between the two hormone levels in the same assay. A high degree of parallelism was confirmed by regression test (*R*^2^ = 0.98, *p* < 0.01).

The assay results for both hormones were expressed as ng/mL for plasma and as pg/mg for hair.

### 2.4. Data Analysis

Descriptive statistics were calculated and expressed as means, standard deviations, and min/max values using the MedCalc Statistical Software version 18.11.3 (MedCalc Software bvba, Ostend, Belgium; https://www.medcalc.org; 2019). In order to evaluate any potential relationships between the analyzed parameters, a non-parametric Spearman rank correlation test was performed. The statistical significance was set at *p* < 0.05 (95% C.I.).

## 3. Results

The results of the descriptive statistics are summarized in Table 1. All the animals were killed between the end of January and the first of March, therefore, according to the reproductive physiology of the species, hinds were approximately at the third to fifth month of gestation. Age ranged from approximately two up to five years. Plasma levels of progesterone ranged from 1.9 to 7.48 ng/mL with a mean of 3.86 ng/mL (SD = 2.14), while plasma cortisol ranged between 0.4 ng/mL and 2.97 ng/mL (mean = 1.44 ng/mL SD = 0.96). On the other hand, the ranges of hair cortisol and P4 were respectively 0.03–0.55 pg/mg (mean = 0.29 pg/mg) and 41.68–153.57 pg/mg (mean = 73.05 pg/mg). 

The Spearman rank correlation analysis, reported in Table 2, did not show any significant result aside from a significant correlation (*p* = 0.042) between hair concentration of P4 and the date of death, and a weak correlation, not statistically significant, between plasma concentrations of P4 and CORT.

## 4. Discussion

As stated in the introduction section, one of the main limitations in studying wildlife reproductive physiology lies within the nature of the sampling possibilities. It is indeed almost impossible to collect repeated samples on the same animal in order to obtain a dynamic profile of specific analytes throughout the different phases of gestation. This is why collecting samples upon killing during the hunting season may represent a good, if not the only, way to perform this kind of research [19]. This study confirms the chance to collaborate with hunters and local biometric centers.

It is widely recognized that analyzing hormones in hair provides information regarding a longer timespan when compared to blood analyses [19,20], but being able to specifically identify such timespan can be challenging. Indeed, due to the nature of the sampling, it is not always possible to completely trim the chosen area to calculate the growth rate of the hair in wildlife animals prior to a subsequent hair sampling. The red deer undergoes two pelages, also known as moultings, one in spring and one in autumn, approximately in May and October, respectively [28,29]. Therefore, the hair collected in the present study belongs to the winter coat and should provide info regarding few months before trimming.

The hormones analyzed in the present study, in both matrices, showed wide ranges suggesting strong individual variations. The differences in progesterone levels may be related to the fact that the sampling took place throughout more than one month and that hinds may be in different phases of pregnancy (approximately from the third up to the fifth month of gestation). Moreover, it was already proved that, in this species, the levels of plasma progesterone fluctuate and correlate with the number of corpora lutea present [14]. On the other hand, cortisol variations, already described in literature for the red deer [20], are related to a multitude of environmental, seasonal, and physiological factors. 

The mean level of blood progesterone recorded in the present study (3.86 ng/mL) is in accordance with previous studies performed in pregnant red deer hinds farmed in paddocks [14,30]. Such agreement of data seems to support the possibility to use data collected from farmed animals also for wildlife. Such statement is extremely preliminary and particular attention will have to be paid if the analytes were to concern stress patterns, which will necessarily be influenced by husbandry conditions and environmental factors. Overall, relatively higher levels of plasma progesterone have to be expected in pregnant hinds when compared to non-pregnant animals, as already reported [31].

The mean hair progesterone level was 73.05 pg/mg with high individual variations most likely explained by the variations detected in plasma levels. As of today, to the best of the authors’ knowledge, no studies were published assessing hair levels of this hormone in red deer and cervids in general. It is therefore quite challenging to discuss such findings. What can be said, on the basis of the present study, is that the analytical approach seems to be reliable and relatively easy to perform. Hair progesterone was assessed in cows in a recent study were no differences between pregnant and non-pregnant animals were found [32]. The authors hypothesized that the cause behind the lack of differences may be the short period between calving and the next successful insemination [32]. Again, it is impossible to compare the results with the ones obtained in the present study in light of the physiological differences between the species and the zootechnical pressure dictated by industry demands.

When compared to progesterone, cortisol is overall more influenced by the conditions in which the animals live [15]. Blood cortisol levels change quickly throughout the day and are therefore relatively unreliable, but quantification in matrices such as feces and hair seems to be a good indicator of chronic stress, potentially also related to the reproductive status of the animal [16,23]. Moreover, cortisol, in all matrices, shows high seasonality variations in red deer [33] as in other wild ungulates [34].

Our results show, as expected, highly variable plasma CORT levels ranging from 0.38 to 2.97 ng/mL. This is likely to be imputed to several “acute” factors and the variability of this hormone pattern itself [22,34]. On the other hand, hair CORT levels are the reflection of the continuous incorporation of the hormone into the hair shaft [15], and thus less influenced by acute stressors. This is why this matrix has been addressed in the last years as highly efficient in wildlife [19]. A study performed on Italian red deer reported mean values of hair CORT of 5.75 pg/mg in females [20], almost 20 times higher than the mean value obtained in the present study (0.29 pg/mg). Factors influencing cortisol production during pregnancy are extremely variable and partially unknown [35]. What is known is that cortisol increases in the late phase of a pregnancy [23], and that, in cows, parturition in the month preceding sampling increases hair cortisol levels [36]. Animals sampled in the present study were approximately at the third month of gestation, and therefore in the first phase of pregnancy. It is possible to suppose that hair levels of CORT would have been higher in case of later phases of gestation. The correlation between hair CORT and chronic rather than acute stress was further confirmed by a study carried out on a red deer population from the central Italian Alps, Sondrio Province [20]. 

Our correlation analysis did not highlight any relationship between the analyzed parameters aside from a significant correlation (*ρ* = 0.659; *p* = 0.042) between hair P4 and the date of death. This correlation may be explained by the ongoing pregnancy, characterized by high levels of P4. Therefore, it is likely that hinds killed later on have had higher accumulation of this hormone in the hair. A weak correlation, not statistically significant, was also found between the plasma levels of the two hormones, P4 and CORT (*ρ* = 0.612; *p* = 0.060). Nonetheless, it is important to acknowledge that the low sample size may be responsible for a weak power of the statistical analysis.

The present study strengthens the use of hair for endocrinological evaluations in wild animals, in light of the easy and non-invasive sampling procedure and the representation of longer time periods in one single sample [15]. Our purpose was to describe the concentration of progesterone and cortisol, from hair and blood, of the wild population of red deer females (*Cervus elaphus* L., 1758) living in the Apennine area, during a particular physiological situation such as gestation. In conclusion, performing such studies on hunted animals seems to be useful in better understanding the physiology of wildlife animals and may provide new useful data that can benefit the species and the scientific community.

## Figures and Tables

**Table 1:** Descriptive data of the red deer hinds and quantified hormones.

Animal	Date of Death	Age (Months)	Plasma (ng/mL)		Hair (pg/mg)	
			P4	CORT	P4	CORT
1	26 January	36	3.23	0.77	19.47	0.50
2	9 February	22	2.20	0.38	41.68	0.05
3	12 February	45	3.15	1.87	50.76	0.23
4	19 February	30	3.17	2.97	96.91	0.16
5	19 February	32	7.44	1.79	153.57	0.36
6	19 February	46	7.48	1.91	31.40	0.21
7	19 February	36	5.44	2.73	69.52	0.55
8	1 March	31	2.51	0.50	104.75	0.51
9	1 March	43	2.09	0.40	80.13	0.03
10	1 March	58	1.90	1.05	82.3	0.28
**mean (SD)**		37.9 (10.3)	3.86 (2.14)	1.44 (0.96)	73.05 (39.85)	0.29 (0.19)
**min–max**		22–58	1.90–7.48	0.38–2.97	19.47–153.57	0.03–0.55

P4: progesterone; CORT: cortisol.

**Table 2:** Spearman rank correlation coefficients (*ρ*) table with *p* values (95% C.I.).

	Plasma CORT	Hair CORT	Plasma P4	Hair P4	Age	Date of Death
**Plasma** **CORT**		0.236 *p* = 0.511	0.612 *p* = 0.060	0.091 *p* = 0.809	0.182 *p* = 0.614	−0.051 *p* = 0.897
**Hair** **CORT**			0.358 *p* = 0.310	0.152 *p* = 0.676	0.030 *p* = 0.934	0.006 *p* = 0.996
**Plasma** **P4**				−0.164 *p* = 0.652	−0.036 *p* = 0.920	−0.380 *p* = 0.277
**Hair** **P4**					−0.261 *p* = 0.466	0.659 *p* = 0.042
**Age**						0.276 *p* = 0.436
**Date of Death**						

Date of Death was reported according to the Julian calendar.
